# Erythema Nodosum Leprosum Reaction Masquerading as Rheumatoid Arthritis

**DOI:** 10.7759/cureus.57312

**Published:** 2024-03-31

**Authors:** Kirti Deo, Aravind Reddy, Rohan Manoj, Shrishti Singh

**Affiliations:** 1 Dermatology, Dr. D. Y. Patil Medical College, Hospital & Research Centre, Dr. D. Y. Patil Vidyapeeth (Deemed to be University), Pune, IND

**Keywords:** mycobacterium leprae, neglected tropical diseases, erythema nodosum leprosum, arthritis, leprosy

## Abstract

Erythema nodosum leprosum is a type 3 hypersensitivity reaction that often presents with transient eruptions of red papules, plaques, and nodules. A 52-year-old female presented with multiple joint pain that was being treated as rheumatoid arthritis (RA), but through clinical examination, she was found to have Hansen's disease with a type 2 reaction. Hence, the importance of a thorough clinical examination is a must for the timely and correct diagnosis of patients suffering from Hansen's disease.

## Introduction

Leprosy is a chronic granulomatous disease caused by the two principal infective agents. Mycobacterium leprae (M. leprae) is the most common causative organism for this disease. Rarely, Mycobacterium lepromatosis has been implicated in a few countries, such as Mexico and Costa Rica [1]. The cardinal signs of leprosy entail a hypopigmented or erythematous patch with hypoesthesia and thickened peripheral nerves [2]. In 2017, there were approximately 200,000 new cases reported worldwide. India, Brazil, and Indonesia account for most new cases (80.2%) [3].

Conventional forms of leprosy can often be diagnosed by a punch biopsy of the lesions and by demonstrating the acid-fast, fuchsia-colored bacilli on a slit skin smear (SSS) examination [3]. Based on the SSS, the bacterial index can be calculated, which is an estimate of the number and dissemination of M. leprae across the body. M. leprae has a prolonged incubation period of approximately three to five years and a slow generation time [3,4]. Although the exact mechanism of transmission is unknown, it is widely accepted that leprosy spreads primarily through the droplet route [1,4]. However, even gastrointestinal, transplacental, and direct inoculation routes have also been avenues of disease transmission [2,4]. The type 2 lepra reaction is a T helper 2 (TH2)-mediated type 3 hypersensitivity reaction. Hence promoting further division and dissemination of bacilli to multiple organs such as the nose, eyes, bones, testes, and the reticuloendothelial system [3,4]. 

Lepra reactions are hypersensitivity phenomena that further complicate the disease by inducing acute neuritis. There are two types of lepra reactions. An inflammatory transformation of the patient’s lesions characterizes type 1 lepra reactions, while type 2 lepra reactions present transient tender nodule lesions [4,5].

## Case presentation

A 52-year-old female patient presented to the hospital with complaints of joint pain for the past 20 months. She was previously diagnosed with rheumatoid arthritis based on the reports of her rheumatoid factor, which showed a weak positive (27.0) and was on treatment for 18 months with low doses of prednisolone and methotrexate that would sufficiently control her symptoms. However, she had developed debilitating arthralgia over the past week after stopping her medication on her own accord. The joint pain was continuous and severe, increasing in intensity in the evenings and during physical activities. This excruciating pain had left her confined to a wheelchair. Two days ago, she noticed a crop of red papules and plaques all over the body, which was associated with febrile episodes the day after. She had even developed three ulcers over the right hand due to some unnoticed trauma. On examination, the multiple tender papules, plaques, and nodules, coupled with the thermal damage-induced digital ulcers, led us to the diagnosis of leprosy in the erythema nodosum leprosum (ENL) reaction. However, we had re-investigated her for rheumatoid factor and anti-cyclic citrullinated peptide (anti-CCP), both of which came back negative.

**Figure 1:**
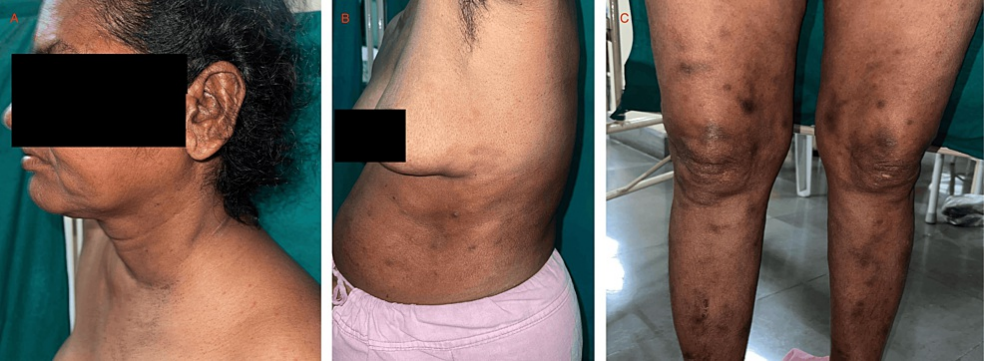
Results of the dermatological examination A: Nodular lesions over the left ear lobe; B: Multiple erythematous and hyperpigmented plaques and nodules over the trunk; C: Multiple erythematous and hyperpigmented nodules over the legs.

**Figure 2:**
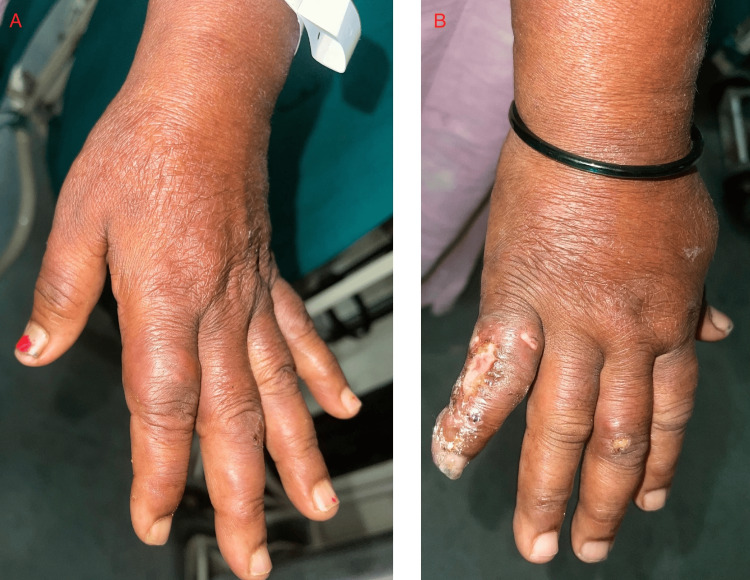
Results of the dermatological examination A: Multiple well-defined red nodules are present over the dorsum of the left hand; B: Three ulcers with sloping edges and pink granulation tissue over the base of the dorsal aspect of the third and fifth digits.

**Figure 3:**
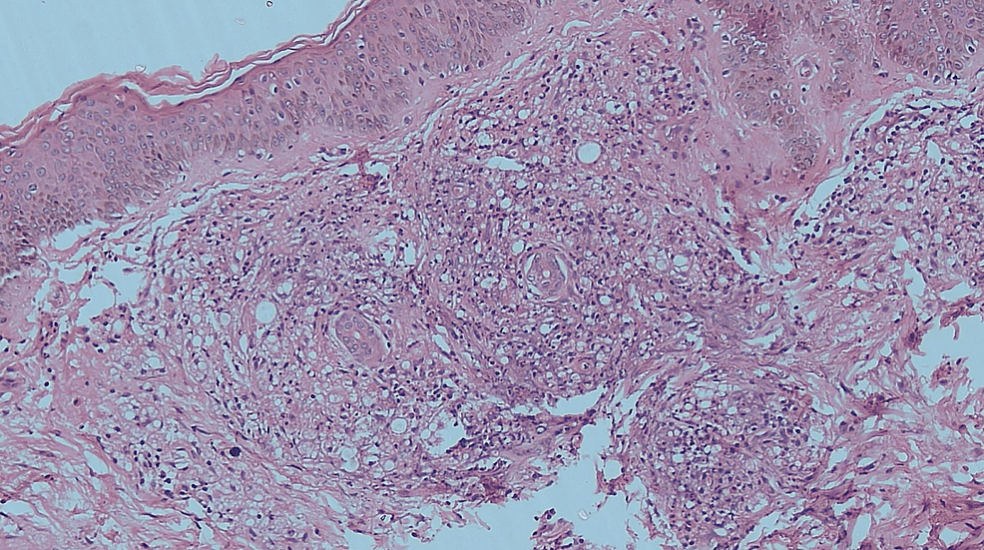
Photomicrograph displays epidermis with flattening of rete ridges The dermis shows diffuse inflammation with neutrophils, foamy macrophages, and a few lymphocytes. A clear Grenz zone is appreciated. Granulomas are absent in this section (H and E x 100).

On inquiry, she gives a history of paresthesia over her hands and feet and recalls multiple instances where her footwear would spontaneously slip away below her feet. She also recalls a few instances from the past year in which she developed burns over her fingers due to her inability to discern the temperature of hot liquids. A detailed neurological examination revealed that the radial and ulnar nerves were tender and palpable in both upper limbs. Temperature and fine touch sensations were impaired in both hands extending from the fingers to the wrist joints and in both feet extending from the toes to the ankle joints. However, there were no gross abnormalities in the motor examination except the reduced range of motion due to the arthralgia. On a dermatological examination, there were multiple erythematous papules and nodules over the upper limbs, trunk, legs, and buttocks, and a nodular infiltration over the earlobe (Figures 1, 2). Three ulcers with sloping edges, pink granulation tissue over the floor, and serous discharge were present on her right hand. The history of tender evanescent lesions along with arthritis and neurological aberrations led us to the diagnosis of borderline lepromatous (BL) Hansen's with erythema nodosum leprosum (ENL) reaction, which was confirmed by a slit skin smear (SSS) and skin biopsy (Figure 3). Shortly after administering a high dose of corticosteroids, she regained full mobility, and the arthralgia had completely ceased overnight. She was counseled regarding the various clinical presentations of her disease and to maintain proper care of the anesthetic limbs. She was discharged shortly after the initiation of multi-bacillary multi-drug therapy (MB-MDT).

## Discussion

ENL, also known as the type 2 lepra reaction, is caused by the tissue deposition of immune complexes against Mycobacterium leprae. It often presents transient eruptions of red papules, plaques, and nodules [2,3]. Constitutional symptoms such as myalgia, fever, and arthralgia often precede the onset of skin lesions. Hence, patients can be erroneously diagnosed with rheumatoid arthritis [2,6]. Rheumatoid arthritis can have numerous extra-articular manifestations, such as erythema nodosum, aortitis, myocarditis, pericardial effusion, stroke, arrhythmias, and congestive cardiac failure. The absence of these features and a negative rheumatoid factor and anti-CCP report helped us rule out the diagnosis of rheumatoid arthritis [7,8]. Furthermore, the SSS and biopsy reports, along with the drastic symptomatic improvement in arthralgia seen immediately after the first dose of corticosteroids, aided us in substantiating the diagnosis of BL Hansen's disease in type 2 reactions. Patients with a high bacillary index have a greater chance of developing ENL reactions [2,4].

ENL can often be the first inkling of the disease because the excruciating pain prompts the patient to seek medical advice. Neuritis, a dreadful complication of lepra reactions, can be clinically demonstrated by tenderness on palpation of peripheral nerves [9]. Permanent nerve damage can be a grave sequela to multiple episodes of neuritis. Hence, early diagnosis and initiation of immunosuppressants can vastly improve clinical outcomes [2,3,4].

Our patient had already presented with features of neuropathy, as evident through the paresthesia over the hands and feet, reduced temperature sensations over the same areas, and the appearance of ulcers following an unknown trauma. Peripheral neuropathy can occur secondary to demyelination following bacillary invasion or due to autoimmune damage due to untreated neuritis [2,3,4]. Trophic ulcers commonly occur secondary to pressure necrosis and autonomic dysfunction. Furthermore, thermal insults to the skin in areas of anesthesia can also lead to necrosis and ulcer formation. Corneal anesthesia predisposes exposure to keratitis and opacities, which can eventually be a cause of blindness. Bacillary invasion and cortical resorption of bones can cause deformities over the toes and fingers, which may impair their occupational opportunities [2,4]. Other features that led to the diagnosis of leprosy were the nodular infiltration of the ear lobe and the appearance of the classical erythematous papules and plaques.

Initially, high doses of oral glucocorticoids, such as prednisolone (1 mg/kg), may be administered and gradually tapered if signs of neuritis are absent [2]. Thalidomide, an immunomodulatory agent, can be co-administered with glucocorticoids to maintain greater disease control [2,4]. Furthermore, the addition of thalidomide can lead to lower doses of glucocorticoids during the maintenance phase of therapy. This will reduce the adverse effects of high doses of corticosteroids, such as electrolyte imbalances, metabolic derangements, hypothalamus-pituitary axis suppression, myopathy, peptic ulcers, etc. [2,4]. Shortly after treating the neuritis, she was administered MB-MDT, which consists of dapsone, clofazimine, and rifampicin, taken over one year [10].

The important aspect of leprosy in reducing the worldwide burden relies on tracing the contacts and starting chemoprophylaxis. Contact tracing increases early case detection and early treatment, breaks the chain of transmission, and prevents disabilities associated with leprosy. Studies show the contacts of leprosy cases are sixfold more likely to develop disease than those in the general population. Contacts with multibacillary (MB) patients have an eight-fold increased risk compared to paucibacillary (PB) contacts [11]. For chemoprophylaxis, current strategies using a combination of Bacillus Calmette-Guerin (BCG) and rifampicin appear to be complementary for protection against the disease. This strategy showed a protective effect of 80% [12]. 

## Conclusions

Erythema nodosum leprosum may initially present with arthralgia prior to the onset of the evanescent papulonodular lesions. Our case highlights why it is essential to look for the cardinal features of leprosy and lepra reactions in patients presenting with arthralgia, especially in leprosy-endemic regions.
